# Methodological Rigor and Integrity of Systematic Reviews and Meta‐Analyses From Nursing‐Affiliated Institutions in North African Countries: The FALCON‐1 Study

**DOI:** 10.1002/cesm.70094

**Published:** 2026-07-30

**Authors:** Nassima Bouzar, Aya Ikhelk, Abdelmounaim Manoussi, Nasser Laouali, Asmaa Habib, Aurélie Vignal, Badia Jabrane, Khalid El Bairi

**Affiliations:** ^1^ Faculty of Medical Sciences, UM6P Hospitals University Mohammed VI Polytechnic Ben Guerir Morocco; ^2^ Laboratoire Éducations et Pratiques en Santé (LEPS, UR 3412) CHU Toulouse, Université Sorbonne Paris Nord Villetaneuse France

**Keywords:** evidence synthesis, integrity, meta‐analysis, methodological rigor, North Africa, nursing sciences, systematic reviews

## Abstract

**Background and Objective:**

Systematic reviews (SRs) and meta‐analyses (MAs) are central to evidence‐based practice (EBP). Despite their growing volume in low‐ and middle‐income countries, especially those produced by nursing‐affiliated institutions in North Africa (NA), no empirical meta‐research has systematically assessed their methodological quality, reporting practices, and research integrity indicators. This study investigated methodological quality, reporting practices, and scientific integrity of SRs and MAs conducted in this context over the past decade.

**Methods:**

We conducted a meta‐research synthesis of SRs and MAs in nursing sciences published between 2015 and 2025 using the PubMed database. Eligible reviews used explicit SR/MA methods, had a first or last author affiliated with a NA institution, and were identified through structured searches. Methodological and reporting quality were assessed using PRISMA and MOOSE guidelines, complemented by an exploratory 10‐point composite quality score. Associations between review characteristics and quality indicators were examined.

**Results:**

We included 38 SRs/MAs, of which 15 included quantitative synthesis. Only 50% reported PROSPERO registration, with several registrations being retrospective or inaccurate. While eligibility criteria, database searches, and quality appraisal were commonly reported, dual data extraction and gray‐literature searches were less common. Validated appraisal tools were inconsistently applied, and reporting guidelines were sometimes inappropriately used. The composite score indicated overall modest methodological quality; with PROSPERO registration was the only factor associated with higher quality scores (*p* = 0.008). Heterogeneity was usually reported, whereas sensitivity analyses and publication‐bias assessments were inconsistently conducted.

**Conclusions:**

This study highlights important methodological and integrity‐related deficiencies in SRs and MAs in NA. Strengthening methodological expertise in this central field of clinical practice is critical.

## 1 Background

Systematic reviews (SRs) and meta‐analyses (MAs) are cornerstones of evidence‐based practice (EBP). They are considered the highest level of evidence in the health sciences and, when rigorously conducted, are pivotal for decision‐making. In clinical practice, EBP aims to combine the best available evidence and professional expertise, while considering the availability of resources and patients' preferences [1, 2]. In the publish‐or‐perish era, systematically appraising the best available scientific evidence and integrating new knowledge into clinical, administrative, or educational practice has become increasingly challenging. Notably, an expanding body of evidence demonstrates that the development of clinical practice guidelines is increasingly challenged by insufficient methodological rigor and the suboptimal quality of published SRs, MAs, and other forms of research synthesis [3, 4] [5] [6]. Most importantly, EBP directly affects care quality by improving treatment protocols and impacting costs; however, poorly conducted or poorly reported research can expose patients and providers to risks. Moreover, research studies with methodological flaws or questionable reporting may compromise the reliability of published findings, potentially distorting the conclusions of SRs and MAs. In response, several tools and guidelines have been developed to strengthen the methodological rigor and reporting quality of these reviews. These include critical appraisal checklists such as the Newcastle−Ottawa scale and ROB‐2 tool, as well as standard reporting guidelines like PRISMA and CONSORT that promote best practices in study reporting [7]. In addition, greater emphasis has been placed on prospective protocol registration in recommended databases such as PROSPERO to enhance transparency, reduce redundancy, and minimize reporting bias. Nevertheless, despite international efforts and recommendations, the meta‐research field still suffers from unnecessary, misleading, or conflicted studies [8, 9].

The methodological quality and integrity of SRs and MAs vary widely across domains and regions [10, 11, 12, 13]. It is hypothesized that SRs and MAs may lack rigor and exhibit methodological flaws, particularly in regions where research infrastructure and training are less developed, such as in low‐ and middle‐income countries (LMICs). On the other hand, EBP is gaining attention in North African (NA) health systems, as these countries are increasingly investing in improving the quality of care for their populations to tackle the dual burden of communicable and noncommunicable diseases. Consequently, the capacity to generate high‐quality evidence should continue to evolve to support decision‐making, particularly in nursing, which has long been regarded as an experience‐based profession, particularly in LMICs where training in EBP remains limited [14, 15].

Little attention has been given to the methodological quality of SRs and MAs in the nursing field, particularly in LMICs. Importantly, LMICs are increasingly recognizing the need to produce local evidence to support patient care and healthcare policy development. Focusing on NA is valuable as research outputs are growing in nursing and allied health sciences from countries such as Morocco, Egypt, and Tunisia [16]. Global evidence synthesis frameworks often underrepresent this region and other LMICs. Thus, critically evaluating the quality of SRs and MAs produced by these countries can help identify both strengths and gaps, offering a clearer picture of the integrity and methodological rigor of the evidence generated in this important field. Importantly, such an appraisal may also support the development of training opportunities and regional guidelines by ensuring that locally generated evidence is better aligned with local health challenges.

In this study, we focused on NA as a representative LMIC region. We assume that, as in many other LMICs, there are limited opportunities for formal training in evidence synthesis [17, 18, 19, 20]. Also, recently published evidence in this field from high‐income countries might not be a suitable proxy for LMICs [21]. These constraints may influence the methodological rigor and reporting quality of SRs and MAs produced by nursing‐affiliated institutes, underscoring the importance of evaluating their integrity and reliability, given the potential direct impact of such research on patient care. Our findings could help guide authors, editors, and academic institutions in strengthening research training, improving the reporting of this important type of publication, and informing the design of future, more robust evidence syntheses.

Our study's primary objective is to evaluate the quality and scientific integrity of SRs and MAs published in the field of nursing sciences in NA. To achieve this objective, we conducted a critical meta‐synthesis of SRs and MAs published over the past 10 years using several quality and compliance criteria. In this paper, we report findings from FALCON‐1, a study within a broader FALCON (*Focused AppraisaL of COmpliance with guideliNes*) research integrity initiative, which examines the quality and integrity of SRs and MAs in clinical and biomedical fields.

## 2 Methods

### 2.1 Study Design and Eligibility Criteria

We conducted a meta‐research synthesis aimed at evaluating methodological quality, reporting practices, and integrity‐related indicators of SRs, with or without MAs over a 10‐year period (January 2015 to September 2025) in the field of nursing sciences in NA. The study included publications affiliated with institutions located in Morocco, Algeria, Tunisia, Libya, Egypt, and Sudan. SRs/MAs were included if they met the following criteria: (a) explicit use of SR methodology (with or without an MA), (b) direct relevance to nursing sciences or an affiliation with a nursing‐related institution. This latter criterion was added because several nursing institutes in this region produce publications outside the strict nursing field, yet these works may still influence clinical decision‐making and public health. We therefore sought to provide a broader picture of the research landscape. (c) The first or last author affiliated with a NA country, and (d) publication between January 1, 2015, and September 15, 2025. Studies were retained only when a clear nursing focus was evident or when published by a nursing affiliation.

Borderline cases were reviewed by two researchers, with inclusion based on consensus. We excluded narrative reviews, other meta‐synthesis‐related articles such as qualitative meta‐syntheses, scoping or rapid reviews, and umbrella reviews, articles without clearly identifiable geographic affiliation; and SRs/MAs in which a NA researcher was limited to co‐authorship positions only. Our study protocol was not prospectively registered. This meta‐research synthesis was primarily descriptive and exploratory in nature, and protocol registration is not yet standard practice for this type of evaluation.

### 2.2 Data Sources, Search Strategy, and Data Extraction

**Figure 1:**
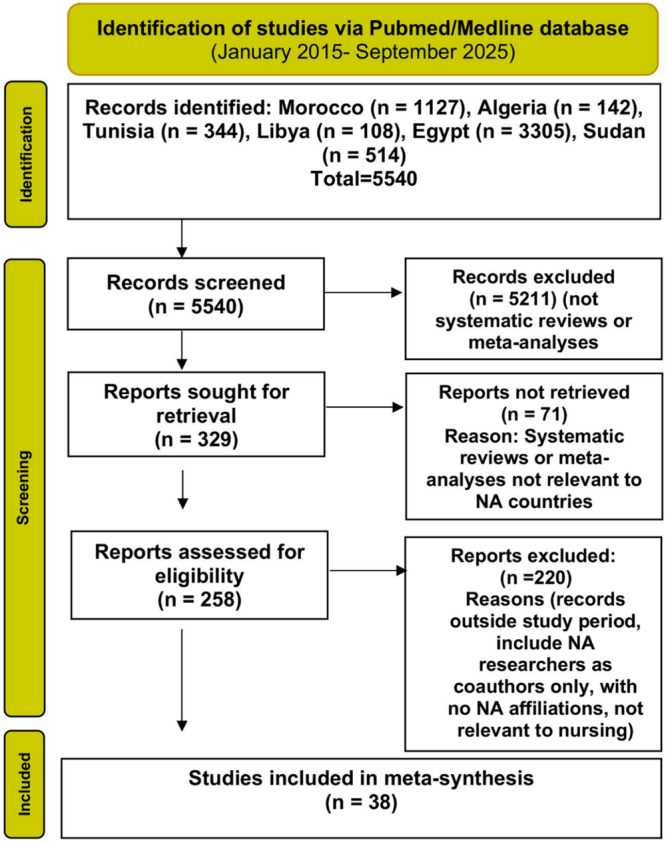
PRISMA‐style flowchart of screened studies. NA, North Africa. *Source:* Page MJ, et al. BMJ 2021;372:n71. doi: 10.1136/bmj.n71. This work is licensed under CC BY 4.0. To view a copy of this license, visit https://creativecommons.org/licenses/by/4.0/.

Articles were identified predominantly through bibliographic searches on PubMed/Medline. The selection of this database was based solely on its ability to provide structured indexing and standardized filters for SRs and MAs, enabling consistent identification of evidence syntheses for meta‐research purposes. Given the methodological focus of this study, comprehensive primary evidence retrieval was not the primary objective. To locate relevant SRs and MAs in the field of nursing sciences in NA, the search strategy used Boolean operators “AND” and “OR” to combine the terms “*nursing”* or “*nurse”* with the names of each NA country (Morocco, Algeria, Tunisia, Libya, Egypt, and Sudan), applying the “Systematic Review” and “Meta‐Analysis” filters. Each result was manually reviewed, article by article, against the inclusion criteria. No language filters were applied. All retrieved records were screened, and data were extracted independently by two reviewers (N.B. and A.K.). A third reviewer (the principal investigator, K.E.B.) conducted a final verification of the extracted data by reviewing all variables and their corresponding entries. Discrepancies were resolved through discussion among the authors and consensus. Reviewers participated in training and extraction sessions prior to data collection to ensure consistency through simulation sessions, piloted on a limited number of articles. Journal quartiles for each year were retrieved from the SCImago and Scopus databases after reviewing the article publication dates and journal name (or its ISSN number). When a journal was assigned to more than one quartile across subject categories, the quartile closest to nursing sciences or to the article's field was selected on a case‐by‐case basis. Although we used structured search and predefined inclusion criteria, this study is neither an SR nor an MA. Instead, it is a meta‐research synthesis focused on the quality of published SRs and MAs, and therefore, effect size estimation and risk‐of‐bias assessment at the primary study level were not applicable. A PRISMA‐style flowchart displaying screened studies is shown in Figure 1.

### 2.3 Evaluation of Methodological Quality, Transparency, and Ethical Elements

Methodological and reporting quality were assessed using predefined criteria informed by established reporting guidelines (PRISMA and MOOSE) and methodological appraisal frameworks (AMSTAR‐2). The following essential variables were evaluated: protocol registration (presence, PROSPERO number, updates), formulation of the research question using the PICO framework, search strategy (eligibility criteria, keywords, databases, gray literature), dual‐reviewer selection and extraction processes, and assessment of the risk of bias in the included studies. For example, regarding the PICOS framework, when research questions were not clearly formulated, we examined the full texts to determine whether the investigators had fully described their aims according to this basis. For MAs, we recorded whether authors assessed statistical heterogeneity using *I* ^2^ index; whether sensitivity and subgroup analyses were conducted when heterogeneous findings were reported; and whether publication‐bias assessment was performed when the number of included studies allowed meaningful evaluation. We also recorded whether the studies declared the use of reporting guidelines (PRISMA, MOOSE, or none), methodological limitations, funding sources, conflicts of interest, and author contributions. We also cross‐checked the author names listed in the included publications and those recorded in the PROSPERO database, as well as the corresponding registration codes, for metadata verification. In addition, we consulted journal publishing policies and author guidelines to verify the requirements for protocol registration for SRs and MAs. To ensure no relevant information about our variables of interest was missing, we also reviewed any supporting materials published by the authors.

We used selected elements of the AMSTAR‐2 tool [22] and an in‐house checklist (Supporting Information Appendix, pp. 1–3) to appraise SRs and MAs. We applied our tailored checklist to examine compliance and methodological rigor, reporting quality, and research integrity in greater detail. A pilot 10‐point scoring system was developed to quantitatively assess the methodological quality of included reviews. Nine items were selected, covering protocol registration and updates (3 points), clear formulation of PICOS question (1 point), dual screening and data extraction (1 point each), gray literature searching (1 point), use of study quality assessment tools (1 point), and transparency indicators encompassing author contributions and conflict of interest declarations (1 point each). MA‐specific parameters, such as heterogeneity variables, were omitted because our review was not limited to MAs, and the number of MAs was small. Quality scores were grouped into low (0−4 points), moderate (5−7 points), or high (8−10 points). The point allocation within the pilot scoring system was based on consensus regarding the relative importance of core methodological and transparency criteria. Because this scoring system is exploratory, it was intended for preliminary use only and will undergo formal validation in the larger upcoming FALCON‐2 study.

### 2.4 Statistical Analysis

After data cleaning, preliminary analyses included transforming only one continuous variable (number of coauthors) into a categorical variable based on the median value. Descriptive statistics were used to summarize extracted data. Categorical variables were presented as frequencies and percentages, and quantitative variables as medians with their interquartile ranges (IQR) after assessing normality using the Shapiro–Wilk test. Fisher's exact test was used to compare categorical variables due to the small sample size and violation of the Chi‐square test assumptions. When the overall Fisher's test was significant, pairwise comparisons across more than two groups were performed with Bonferroni correction for multiple comparisons. These analyses were applied only to quality‐score categories. Nonparametric Spearman's rank correlation was also computed to investigate the relationship between quality scores and selected variables, including the number of coauthors, after verifying assumptions.

Comparisons across groups, particularly the impact of PROSPERO‐related information on quality scores, were pre‐planned using non‐parametric tests adapted to small samples, while additional association and correlation analyses were conducted in an exploratory manner. Missing data were reported in tables and footnotes for each variable when present. Therefore, a census‐based approach was implemented by including all available cases during the study period. All statistical tests were two‐tailed, with a significance level of *α* = 0.05. Analyses were conducted on a sample of 38 observations. All statistical analyses were performed on IBM SPSS Statistics for Windows, Version 25 (IBM Corp., Armonk, NY). Statistical analyses and reporting were performed according to SAMPL guidelines [23].

## 3 Results

### 3.1 Description of Included Publications

A total of 38 SRs and MAs were included (Table 1). More than half (60.5%, *n* = 23) were SRs without MAs, while 39.5% (*n* = 15) combined SR methods with quantitative pooled synthesis, including one network MA. Nearly half of the reviews (47.4%, *n* = 18) included observational studies alone, followed by mixed‐design studies (39.5%, *n* = 15). Reviews of interventional studies (*n* = 2; 5.3%), educational videos (*n* = 1; 2.6%), and preclinical evidence (*n* = 1; 2.6%) were less common. Most of the publications were in the disciplines of nursing sciences (*n* = 16, 42.1%). This was followed by public health (*n* = 10, 26.3%), epidemiology (*n* = 4, 10.5%), and psychology (*n* = 3, 7.9%). The remaining five publications (13.2%) were classified under other fields.

**Table 1:** General features of included SRs and MAs (*n* = 38).

Characteristic	*N* (%)
Publication type	
Systematic review without meta‐analysis	23 (60.5)
Systematic review with meta‐analysis	15 (39.5)
Type of included studies	
Observational studies	18 (47.4)
Mixed studies	15 (39.5)
Interventional studies	2 (5.3)
Educational videos	1 (2.6)
Not reported/difficult to assess	1 (2.6)
Preclinical	1 (2.6)
Number of included original studies^β^	
Median (IQR; min−max): 24 (32; 5−153)	
Subject enrollment^π^	
Median (IQR; min−max): 5528 (110,483; 45−862,037)	
Publication period/year	
2025	14 (36.8)
2024	9 (23.7)
2023	4 (10.5)
2016−2022	11 (20)
Research discipline	
Nursing sciences	16 (42.1)
Public health	10 (26.3)
Epidemiology	4 (10.5)
Psychology	3 (7.9)
Other fields^Ω^	5 (13.2)
Publication country	
First author	
Morocco	21 (55.3)
Egypt	5 (13.2)
Tunisia	3 (7.9)
Sudan	2 (5.3)
Other^∑^	7 (18.4)
Last author (principal investigator)	
Morocco	20 (52.6)
Egypt	6 (15.8)
Tunisia	2 (5.3)
Libya	1 (2.6)
Other^¥^	9 (23.7)
Number of co‐authors, median (IQR; min−max): 4 (4; 1−12)	
Article relevance to nursing fields	
Yes	17 (44.7)
No	21 (55.3)
Journal relevance to nursing fields	
Yes	13 (34.2)
No	15 (65.8)
Journal quality based on Scopus quartiles	
Q1	19 (50)
Q2	10 (26.3)
Q3	7 (18.4)
Q4	1 (2.6)
Not applicable^£^	1 (2.6)
Gender of publication authors	
First author	
Women	21 (55.3)
Men	17 (44.7)
Last author (principal investigator)	
Men	24 (63.2)
Women	14 (36.8)
International collaboration	
Present	19 (50)
Absent	19 (50)

*Note:* ^∑,¥^Other countries rather than those specified in the inclusion criteria (detailed in Supporting Information Appendix pp 4). ^β^Calculated using sample size of 37 articles because one publication included videos not real studies. ^π^Conducted on a sample size of 23 articles because of missing data and estimation of total enrollment was not possible. ^£^Not indexed in the Scopus database. ^Ω^Health education (*n* = 2), neuroscience (*n* = 1), nutrition (*n* = 1), infectious disease (*n* = 1). For all journals in which the selected articles were published, please refer to Supporting Information Appendix pp 4−5.

Among 37 SRs and MAs reporting extractable data, the median number of original studies included was 24 (range: 5−153). Additionally, 23 SRs and MAs provided information regarding total subject enrollment, with a median of 5528 participants (IQR/range: 110,483/45−862,037). Regarding authorship, first authors were most commonly affiliated with Morocco (55.3%), followed by Egypt (13.2%), Tunisia (7.9%), and Sudan (5.3%). The last (senior) authors showed a similar geographic pattern, with Morocco again being the most represented (52.6%), followed by Egypt (15.8%), Tunisia (5.3%), and Libya (2.6%). The median number of co‐authors per publication was 4 (range: 1−12). Only 44.7% (*n* = 17) of the reviews were directly relevant to nursing, whereas 55.3% (*n* = 21) did not explicitly target nursing topics. Similarly, only 34.2% (*n* = 13) were published in journals relevant to the nursing discipline; the remainder appeared in journals outside the nursing domain. Based on Scopus indexing, half of the journals were classified in Q1 (50%), followed by Q2 (26.3%), Q3 (18.4%), and Q4 (2.6%), while one journal (2.6%) was not indexed in Scopus. Female authors were more frequently first authors (55.3%), whereas men more commonly appeared as principal investigators (63.2%). Finally, international collaboration was present in half of the included reviews.

### 3.2 Protocol Registration, Compliance, and Transparency Indicators Across Included Publications

Among the 38 included SRs and MAs (Table 2), half (*n* = 19, 50%) reported registering their review on PROSPERO, reflecting only partial commitment to recommended prospective protocol‐registration practices. None of the SRs or MAs that were not registered in PROSPERO used an alternative preregistration registry. Among claimed registrations, only 3 (16%) had updated records following project progress or protocol changes, and most still had an “ongoing” status despite having already been published. In two SRs, the authors provided incorrect registration numbers, including one review for which no PROSPERO registration could be identified. Nearly all registered reviews provided the registration number in their publication (*n* = 18, 95%). Only 10 studies (26.3%) were published in journals whose author guidelines explicitly required PROSPERO registration. One unusual case involved authors who used the registration details of a different review conducted by researchers in another country with a similar ongoing project. Irregularities in authorship were also observed. For example, one SR listed only a single author on PROSPERO, yet the final published version included several authors. The median time from PROSPERO registration‐to‐journal submission interval was 242 days (range: 3−983 days), and the median manuscript submission‐to‐journal acceptance interval was 134 days (range 13−452 days). This suggests that in some cases registration occurred near manuscript submission, raising concerns about retrospective registration. Reporting of other transparency elements was good overall. Most reviews included author contribution statements (*n* = 31, 81.6%), funding acknowledgments (*n* = 31, 81.6%), and almost all of them disclosed conflicts of interest (*n* = 36, 94.7%).

**Table 2:** PROSPERO compliance, other registration practices, and transparency in included SRs and MAs (*n* = 38).

Characteristic	*N* (%)
PROSPERO registration claimed by authors	
Yes	19 (50)
No	19 (50)
Verification of claimed PROSPERO registration	
Verified and confirmed	19 (100)
Not applicableα	19 (0)
Registration updated on PROSPERO?	
Yes	3 (16)
No	16 (84)
PROSPERO registration number	
Provided	18 (95)
Not provided	1 (5)
PROSPERO registration requirement by target journals	
Required in instructions to authors	10 (26.3)
Not required in instructions to authors	28 (73.7)
PROSPERO registration‐to‐journal submission interval (days)µ	
Median (IQR; min−max): 242 (679; 3−983)	
Journal submission‐to‐journal acceptance time (days)Ω	
Median (IQR; min−max): 134 (177; 13−452)	
Author contributions statement	
Provided	31 (81.6)
Not provided	7 (18.4)
Funding acknowledgment	
Yes	31 (81.6)
No	7 (18.4)
Conflicts of interest declaration	
Yes	36 (94.7)
No	2 (5.3)

^α^ These are systematic reviews and meta‐analyses that were not registered on PROSPERO.

^µ^ Calculated using a sample of 17 articles where information on PROSPERO submission and acceptance dates was available.

^Ω^ Calculation made on a sample of 34 articles where information on journal submission to acceptance was available.

### 3.3 Quality Features of Included SRs and MAs

Across the included SRs and MAs, most demonstrated good adherence to established reporting and methodological standards (Table 3). Indeed, two‐thirds clearly presented research questions structured according to the PICO framework (65.8%). Nearly all studies reported eligibility criteria (97.4%) and described their keyword combinations (97.4%) as part of the search strategy, and the majority appropriately selected bibliographic databases for literature search (94.7%). However, only 60.5% reported searching gray literature. The number of bibliographic databases consulted had a median of four (range: 1−11). Most reviews reported conducting the literature search with two independent reviewers (81.6%), though fewer applied dual‐reviewer data extraction processes (60.5%). Most studies performed a formal assessment of primary study quality (86.8%), and among these, 91.9% applied appropriate appraisal tools. Notably, some anomalies were identified regarding the tools used. Indeed, several reviews incorrectly used reporting guidelines, such as CONSORT and STROBE, instead of critical appraisal checklists, resulting in inappropriate quality assessment. Reporting guideline compliance was high overall, with 89.5% explicitly referencing PRISMA in their methodology.

**Table 3:** Reporting and quality features of reviewed SRs and MAs.

Characteristic	*N* (%)
Research question well stated according to PICO	
Yes	25 (65.8)
No	13 (34.2)
Search strategy	
Eligibility criteria	
Yes	37 (97.4)
No	1 (2.6)
Keyword combinations	
Yes	37 (97.4)
No	1 (2.6)
Bibliographic database selection	
Yes	36 (94.7)
No	2 (5.3)
Searching gray literature	
Yes	23 (60.5)
No	14 (36.8)
Not applicableΩ	1 (2.7)
Literature search by two reviewers	
Yes	31 (81.6)
No	7 (18.4)
Number of bibliographic databases used	
Median (IQR; min−max): 4 (2; 1−11)	
Data extraction by two reviewers	
Yes	23 (60.5)
No	15 (39.5)
Study quality assessment	
Yes	33 (86.8)
No	5 (13.2)
Study quality assessment tool appropriate?┼	
Yes	30 (90.9)
No	3 (9.1)
Reporting guidelines used?	
PRISMA	34 (89.5)
Not reported	4 (10.5)
Study limitations statement	
Yes	31 (81.6)
No	7 (18.4)

^Ω^ One SR included videos only.

^┼^ Calculated based on studies that assessed quality (*n* = 33).

Among examined features (Table 4), prior PROSPERO protocol registration was the only factor found to be associated with the quality of SRs and MAs (*p* = 0.008). Overall, reviews with registered protocols consistently demonstrated superior quality scores. Nearly 89% of low‐quality reviews did not have PROSPERO registration. However, Bonferroni‐adjusted pairwise comparisons for individual categories did not reach significance (*p* > 0.05). International collaboration—including with researchers from high‐income countries—showed no significant relationship with methodological quality (*p* = 0.77). Journal‐related quality characteristics, such as Scopus quartiles, were also not associated with differences in methodological quality. Indeed, reviews published in higher‐ranked journals (Q1−Q2) and those published in lower‐ranked journals (Q3−Q4) did not differ significantly (*p* = 0.25). Similarly, publishing in a nursing‐specialized journal was not associated with quality outcomes (*p* = 0.23). Authorship characteristics, including the number of authors and gender of first and last authors, also showed no statistically significant associations (*p* = 0.88, *p* = 0.21, and *p* = 0.58, respectively).

**Table 4:** Association between publication characteristics and methodological quality.

Features	Quality score categories			*p* value
	Low (%)	Moderate (%)	High (%)	
Prior protocol registration				
Yes	11.1^a^	56^a,b^	100^b^	0.008
No	88.9^a^	44^a,b^	0^b^	
International collaboration				
Yes	55.6	52	25	0.77
No	44.4	48	75	
International collaboration with authors based in high‐income countries				
Yes	44.4	48	25	0.88
No	55.6	52	75	
Journal quality based on Scopus quartiles				
Q1−Q2	75	84	50	0.25
Q3−Q4	25	16	50	
Journal specialization in nursing				
YesΩ	22.2	44	0	0.23
No	77.8	56	100	
Number of authors per article‡				
< 4	44.4	36	25	0.88
≥ 4	55.6	64	75	
Gender of first author				
Female	77.8	52	25	0.21
Male	22.2	48	75	
Gender of last author				
Female	22.2	40	50	0.58
Male	77.8	60	50	

*Note:* a,b: Pairwise comparisons of column proportions were conducted using Bonferroni correction. Subscript letters denote subsets whose proportions do not significantly differ at the 0.05 level. Columns sharing the same letter are statistically equivalent.

^Ω^ Based on a sample of 37 SRs and MAs, one journal was not indexed in the Scopus database.

^‡^ A Spearman's correlation showed that the relationship between the number of coauthors and score category was positive but weak and not statistically significant (correlation coefficient = 0.20, *p* = 0.22).

### 3.4 Statistical Heterogeneity and Publication Bias Assessments

The evaluation of statistical heterogeneity and publication bias was inconsistently reported across the reviewed MAs (*n* = 15) (Table 5). Most reviews (12/15, 80%) used a random‐effects model, whereas only one review each (6.7%) applied a fixed‐effect model—despite the absence of heterogeneity among included studies—or a combination of both, and one review (6.7%) used a network MA. Almost all MAs assessed statistical heterogeneity using the *I* ^2^ statistic (14/15, 93.3%). Among the 14 reviews that reported heterogeneity, 11 (78.6%) investigated potential sources of heterogeneity. Sensitivity analyses were performed in less than half of these reviews (5/11, 45.5%), while subgroup analyses were more commonly conducted (10/11, 90.9%). Assessment of publication bias was reported in two‐thirds of the MAs that were eligible for such evaluation (10/14, 66.7%), and study limitations were reported in 81.6% (*n* = 31) of reviews.

**Table 5:** Statistical heterogeneity and publication bias assessment of reviewed MAs (*n* = 15).

Characteristic	*N* (%)
Meta‐analysis model used	
Random effect model	12 (80)
Fixed effect model	1 (6.7)
Both¥	1 (6.7)
Not applicableπ	1 (6.7)
Statistical heterogeneity assessment (based on *I* ^2^)	
Yes	14 (93.3)
No	1 (6.7)
Source of heterogeneity evaluation (*n* = 14)	
Yes	11 (78.6)
No	3 (21.4)
Sensitivity analysis (*n* = 11)	
Yes	5 (45.5)
No	6 (54.5)
Subgroup analysis	
Yes	10 (90.9)
No	1 (9.1)
Publication bias assessment (*n* = 14)	
Yes	10 (66.7)
No	4 (33.3)

^¥^ Depending on the primary comparisons.

^π^ Network meta‐analysis.

## 4 Discussion

SRs and MAs occupy a central place in the hierarchy of scientific evidence. As syntheses of primary studies, they have a considerable impact on clinical practice, public health policies, and the training of healthcare professionals including nurses. It is therefore essential to conduct a rigorous assessment of these publications to strengthen the consistency and relevance of EBP approaches. Various critical reviews have highlighted frequent methodological concerns, including the absence of protocol preregistration and updating, selection bias in primary studies, and a lack of transparency. In the NA context, where nursing research is still developing, evaluating the methodological quality of such syntheses goes beyond a critical review. It also represents a strategic opportunity to propose concrete areas for improvement, to ensure credibility, reproducibility, and applicability of the results produced in daily clinical practice.

Notably, our study showed only partial commitment to prospective protocol registration among researchers in NA. Only a few SRs and MAs (3 out of 19) updated their protocols in the PROSPERO registry. This supports the broader global concern that PROSPERO registration is sometimes used merely to satisfy journal requirements [24, 25, 26]. This is further supported by our findings that only 10 studies were published in journals that explicitly require protocol registration. Lack of awareness among researchers about the importance of protocol registration [27] may also explain why half of the SRs and MAs we analyzed did not include registration information. Therefore, journals, editors, reviewers, and training programs in evidence synthesis should require protocol registration, given its positive impact in generating high‐quality evidence to be used for EBP. Strict peer review that verifies PROSPERO‐related information provided by authors of SRs and MAs should also be standard practice by academic journals. Some authors may use protocol information from similar reviews, reuse registration numbers of similar reviews from other researchers, provide incorrect random numbers, or register their protocol only a few days before submission, as we observed in four of the publications analyzed in our study. Furthermore, the fact that most SRs and MAs included were published in Q1/Q2 journals despite methodological flaws raises concerns about the quality of peer review. Whether indexing databases should incorporate an assessment of peer review quality rather than relying mainly on citation‐based metrics to categorize journal quality is another issue that deserves discussion within the scientific community.

Higher‐ranked journals do not necessarily enforce stricter methodological standards, and lower‐ranked or specialized journals are not inherently lower quality in their publication practices for SRs/MAs. This is supported by emerging evidence suggesting that the majority of SRs published in high‐impact journals did not register their protocols [28]. Similarly, journal impact factor was not found to be associated with study results or methodological quality [29]. To date and to the best of our knowledge, the quality of published research based on Scopus quartiles has not been thoroughly investigated. However, a recent SR and MA demonstrated a positive correlation between the Web of Science impact factor metric and Scopus CiteScore [30].

Moreover, initiatives promoting open science—such as PROSPERO—and the considerable rise of open‐access journals and other open platforms and frameworks may not be sufficient to achieve the goal of improving research quality, as shown in our study and by other authors [31, 32], including in nursing science [33]. Yet, open access platforms like PROSPERO are essential for evaluating quality, as all components of the research process are available for appraisal, as demonstrated in our study, which in turn promotes responsible research. It is noteworthy that a substantial number of SRs and MAs are registered [34], and this has been associated with enhanced methodological quality, as shown in an analysis of more than 180 studies [35]. Some authors also argue that PROSPERO protocols may be copied and used by other researchers while original authors are still working on their projects, and that some reviews may be registered but never completed, as we observed in our study, potentially discouraging others from pursuing the same topic [36]. Although this type of initiative is not perfect, it ultimately depends on the integrity and honesty of researchers, particularly in a field such as EBP, which directly impacts human health. Importantly, it is advised that protocol registration should not be considered a static administrative step, but rather a tool to enhance transparency and quality and to reduce bias. Therefore, developing a positive attitude toward registration in PROSPERO or similar online registries is an unmet need. Our inferential analysis showed that prior PROSPERO protocol registration was the only feature significantly associated with better SR/MA quality score. This suggests that it promotes methodological rigor by following a predefined plan, reducing the risks of selective reporting, methodological drift, and bias compared with low‐quality reviews, which rarely had registered protocols. However, the lack of significant Bonferroni‐adjusted pairwise comparisons between score categories suggests that our meta‐synthesis was underpowered. A larger sample—by including more SRs and MAs—will be necessary before drawing conclusions.

Gender patterns among authors revealed that women were often first authors, whereas men more frequently held senior authorship positions as principal investigators. This aligns with recent evidence suggesting that women are more likely to be first rather than last authors in meta‐research studies [37] and continue to face barriers to attaining more senior positions [38]. Several theories, nevertheless, associate gender diversity with better research quality. Our analysis did not provide sufficient evidence to determine whether author gender is associated with academic output. Although some theories link gender to higher research quality, our analysis did not support this, suggesting that methodological quality was not associated with author gender in our sample. Likewise, team size did not predict better quality scores. This contradicts the common expectation that larger, mixed‐gender teams promote more rigorous, novel, and impactful research [39] through additional layers of review. This suggests that methodological quality depends more on training, experience, and adherence to protocols.

Another key observation from our study was that more than half of the SRs/MAs did not focus on nursing topics and were published outside nursing journals, indicating a potential disconnection from nursing practice needs. This can be explained by the fact that many nursing institutions, particularly in Morocco, lack qualified academic human resources in the field of nursing. As this discipline is only beginning to emerge as an independent field, most local researchers were trained in other areas such as life sciences and related disciplines.

Notably, our analysis showed misuse of reporting guidelines (e.g., CONSORT, STROBE) instead of proper critical appraisal tools, indicating methodological misunderstanding in some reviews. This result aligns with the study by da Costa et al. showing that STROBE recommendations are frequently used inappropriately in SRs and MAs as an instrument to measure methodological quality [40] and with reports by other authors on the misuse of other checklists [41, 42]. This practice may stem from limited training in epidemiological research and from gaps in editorial and peer review control. One could also argue that reporting guidelines are more accessible and visible than critical appraisal tools, which may mislead authors with little or no formal training. Our analysis showed that around 40% of SRs/MAs did not report searching gray literature. While this does not necessarily indicate publication bias, it may increase the risk of missing unpublished or non‐indexed evidence. Gray‐literature searching is a core expectation of rigorous review methods. The exclusion of gray literature from MAs can lead to exaggerated estimates [43]. However, recent evidence also suggests that including gray literature rarely impacted the results and conclusions of MAs [44].

Our findings regarding statistical heterogeneity and publication bias assessments are consistent with patterns widely reported in methodological reviews of MAs quality [45]. Most reviews relied on random‐effects models, reflecting common practice, even when statistical heterogeneity is low. In fact, although the two models use many of the same formulas to calculate statistics, they are not interchangeable [46]. Each model reflects a distinct assumption about the underlying data. Choosing the correct model is therefore essential, both for obtaining accurate estimates and for appropriately framing the analysis [46]. Although most reviews explored heterogeneity, sensitivity analyses were underused while subgroup analyses were far more common as recommended in the Cochrane Handbook for the conduct of SRs and MAs [47]. Finally, publication bias was assessed in about two‐thirds of eligible reviews, mirroring the incomplete reporting [48].

Our analysis also showed that dual‐reviewer screening for eligibility was common, but fewer SRs and MAs used independent dual‐reviewer data extraction, leaving room for data‐extraction errors. Data extraction by two independent reviewers increases the chance of finding discrepancies [49]. Thus, training investigators to accurately review selected studies during data extraction and to pilot this process is important [49, 50], yet it is often inadequately reported in reviews. For example, in our study, three reviewers independently examined the data extraction process, which enabled us to effectively identify errors and discrepancies among authors.

Although international collaborations, especially with researchers from high‐income countries, are often assumed to bring more methodological expertise, our study found no significant association. In fact, one recent study conducted by Thelwall et al. on the relationship between international collaboration and research quality in the United Kingdom found that such collaboration generally leads to higher‐quality research—but only when it involves partners from high‐research‐expenditure economies [51]. In contrast, collaborations with lower research‐expenditure economies were associated with lower research quality [51]. Team diversity has been shown to be beneficial in various scenarios. Accordingly, international research teams may gain from a broader range of ideas, skills, expertise, resources, scientific traditions, and background knowledge [52]. Research from the Global South may be viewed as lower quality from a Global North evaluation standpoint, potentially due to known factors such as weaker research training and reduced institutional support. International partnerships alone do not guarantee methodological rigor; quality appears to be more closely tied to SR processes than to team composition, and it is also possible that these collaborations did not include meta‐research methodology specialists.

Poorly conducted or sub‐optimally reported SRs and MAs can lead to incomplete syntheses, compromise the reliability of clinical protocols, and potentially endanger patient safety. Therefore, the critical appraisal of generated evidence is of paramount importance. Our study, which may reflect broader patterns across the nursing field, sends a message to the scientific community about the need to increase awareness of this issue, which affects a key area of clinical practice. Indeed, suboptimal SRs/MAs can influence nursing education, clinical protocols, and local guideline development.

Our observations raise questions about the actual impact of these publications and highlight the need to strengthen the methodological rigor and clinical relevance of future reviews. Several recommendations for future authors, reviewers, editors, and integrity guidelines developers can be drawn from this meta‐research synthesis. For researchers, it is essential to prioritize transparent protocol development, registration, and updates in leading registries such as PROSPERO. It is also advised not to rely on journal prestige or team composition as substitutes for methodological rigor and reproducibility. Journal editors and reviewers should consider mandatory protocol registration to improve consistency and transparency and to strengthen peer review as a backbone of science. Institutions and educators should support meta‐research investigators through high‐quality methodological training and promote quality over quantity.

Our study has a number of limitations. First, the research was limited to NA, which may restrict the regional and global generalizability of the conclusions. Furthermore, the sample remains small, which reduces statistical power to detect small‐to‐moderate associations, and results should therefore be interpreted with caution. Nonsignificant associations found should be considered inconclusive. Moreover, although no language filters were used, the choice of the PubMed database inherently favors English‐indexed content, which may have led us to miss some eligible studies. The literature search was restricted to a single database, which may have resulted in the omission of eligible studies indexed elsewhere and may reduce the representativeness of NA evidence syntheses. Researchers from NA institutions might perceive this study as a negative evaluation. Our aim, however, was to frame it as a constructive, capacity‐building critical analysis.

## 5 Conclusion

Overall, the appraised SRs and MAs showed acceptable reporting in several areas, but specific methodological and reporting practices require strengthening to ensure reliability. These findings raise important questions about peer‐review standards in medical and nursing journals. These findings underscore the need for larger‐scale studies and further work to strengthen the methodological foundations of nursing research and ensure its impact on practice. There is an urgent need to embed evidence‐based appraisal and evidence synthesis methods in NA nursing programs. Doing so will strengthen the capacity of nurse‐researchers and improve the quality and reliability of SRs and MAs in the region, thereby supporting better evidence‐based healthcare.

## Author Contributions

Conception and design: Khalid El Bairi. Collection and assembly of data: Khalid El Bairi, Aya Ikhelk, and Nassima Bouzar. Data analysis and interpretation: Khalid El Bairi and Nassima Bouzar. Study supervision and review: Khalid El Bairi, Aurélie Vignal, Asmaa Habib, Nasser Laouali, Abdelmounaim Manoussi, and Badia Jabrane. Manuscript writing: Khalid El Bairi and Nassima Bouzar. Final approval of the manuscript: All authors. Accountable for all aspects of the work: All authors.

## Funding

The authors have nothing to report.

## Conflicts of Interest

K.E.B. reports receiving fees and honoraria from NCODA, Techspert, Elsevier, and Springer, and publishing grants from the Cancer Research Institute (Morocco) (not related to this project). B.J. is the pedagogical director of the institute where this study was conducted. The other authors declare no conflicts of interest.

## Supporting information


Supporting File

## Data Availability

The data set of this research is available upon reasonable request from the corresponding author.
