# Supplementary material for: Methodological Rigor and Integrity of Systematic Reviews and Meta‐Analyses From Nursing‐Affiliated Institutions in North African Countries: The FALCON‐1 Study
Source: Cochrane Evid Synth Methods. 2026 Jul 30;4(5):e70094. doi: 10.1002/cesm.70094 (PMC13431894; doi:10.1002/cesm.70094)
Supplement: Supplementary file 1 — Supporting File [file CESM-4-e70094-s001.docx]

**Supplementary Material for :**

**Methodological Rigor and Integrity of Systematic Reviews and Meta-analyses from Nursing-affiliated Institutes from Low- and Middle-Income Countries: The FALCON-1 Study**

**Nassima Bouzar^1^, Aya Ikhelk^1^, Abdelmounaim Manoussi^1^, Nasser Laouali^1^, Asmaa Habib^1^, Aurélie Vignal^2^, Badia Jabrane^1^, Khalid El Bairi^1^**

1. Faculty of Medical Sciences, UM6P Hospitals, University Mohammed VI Polytechnic, Ben Guerir, 43150, Morocco

2. Université Sorbonne Paris Nord – Laboratoire Éducations et Pratiques en Santé (LEPS, UR 3412) CHU Toulouse, France

**Supplementary Table 1. Variables, scoring, and justification of in-house developed checklist**

| **Quality variables** | **Total points** | **Recommended by PRISMA** | **Recommended by AMSTAR-2** | **Justification of variable inclusion** | **Observations/details from our analysis** |
| --- | --- | --- | --- | --- | --- |
| **PROSPERO (or other database) registration: 1 point** (yes vs. no) | 3 points | Yes | Yes | -Required to ensure transparency, reproducibility, and credibility; to prevent redundancy; to promote research integrity; and to reduce the risk of bias, including selective outcome reporting bias. | -One PROSPERO-registered SR was downgraded to 0 because the protocol was registered only a few days before manuscript submission to the journal.  -One PROSPERO-registered SR was downgraded to 0 because the authors used a PROSPERO registration code belonging to another team from another country with a similar research question. |
| **PROSPERO updates: 2 points** (yes vs. no) |  | No | No | -Updating the PROSPERO protocol shows that investigators are conducting the review seriously, not just seeking an approval code for journal requirements.  -Given its significance in research rigor and credibility, it was given 2 points compared to other variables |  |
| **Clear PICO question** (yes vs. no) | 1 point | Yes | Yes | -Essential when planning and reporting the findings because it provides the foundation for methodological rigor, clarity, and interpretability. | None |
| **Comprehensive screening strategy by two reviewers** (yes vs. no) | 1 point | No | Yes | -Essential as it reduces bias, improves accuracy, and ensures that all relevant studies are consistently identified and selected. | None |
| **Data extraction by two reviewers** (yes vs. no) | 1 point | No | Yes | -Minimizes errors and bias, ensuring that key study information is captured accurately and consistently. | None |
| **Search of the Grey literature** (yes vs. no) | 1 point | Yes | Yes | -Helps capture unpublished or non-peer-reviewed studies, reducing publication bias and ensuring a more complete and balanced evidence base. | None |
| **Study quality assessment** (yes vs. no) | 1 point | Yes | Yes | -Identifies the risk of bias within included studies, ensuring that the evidence is interpreted appropriately and objectively. | Three SRs that used quality assessment tools were given a score of 0 because they applied them incorrectly (misuse of reporting guidelines instead of quality assessment tools, use of tools adapted to observational studies for interventional studies, etc.). |
| **Declaration of authors’ contribution** (yes vs. no) | 1 point | Yes | No | -Ensures transparency in who performed each task, promotes accountability, and helps prevent issues such as ghost authorship or undeserved authorship.  -This strengthens the integrity and credibility of SRs/MA. | None |
| **Declaration of authors conflicts of interest** (yes vs. no) | 1 point | Yes | Yes | -It promotes transparency and helps readers assess whether any personal or financial relationships may have influenced the research.  -This strengthens the trustworthiness and credibility of the SR/MA and its conclusions. | None |

**Supplementary Table 2. Countries not meeting inclusion criteria for papers in which the first or last author had a corresponding author from an included country.**

| **Variables** | **N** |
| --- | --- |
| **Other countries in last author position** (total =9)  Saudi Arabia  United Kingdom  United Arab Emirates  Lebanon, Saudi Arabia, Jordan | 4  3  1  1 |
| **Other countries in first author position** (total =7)  United Kingdom  China  United States  Saudi Arabia | 2  1  1  3 |

**Supplementary Table 3.** Detailed list of journals for the included publications

| **Journal names (abbreviated)** | **N** |
| --- | --- |
| Anesth Crit Care Pain Med  Arch Dermatol Res  Biomark Insights  BMC Infect Dis  BMC Nephrol  BMC Nurs  BMC Public Health  Br J Nurs  Br J Nutr  Curr Oncol  Future Sci OA  Hum Resour Health  Int J Environ Res Public Health  Int J Nurs Pract  Interdiscip Perspect Infect Dis  J Clin Nurs  J Educ Eval Health Prof  J Nurs Scholarsh  J Parasit Dis  JMIR Mhealth Uhealth  Korean J Med Educ  Medicine (Baltimore)  Metab Syndr Relat Disord  Midwifery  Nurs Rep  Nutrients  Osong Public Health Res Perspect  PLoS One  Rev Epidemiol Sante Publique  Semin Arthritis Rheum  Soc Psychiatry Psychiatr Epidemiol  Syst Rev  Turk J Pediatr  Womens Health (Lond) | 1  1  1  1  1  1  1  1  1  1  1  1  1  1  1  2  2  1  1  1  1  1  1  1  1  1  2  2  1  1  1  1  1  1 |
